# The LuxI/LuxR-Type Quorum Sensing System Regulates Degradation of Polycyclic Aromatic Hydrocarbons via Two Mechanisms

**DOI:** 10.3390/ijms21155548

**Published:** 2020-08-03

**Authors:** Zhiliang Yu, Zeyu Hu, Qimiao Xu, Mengting Zhang, Nate Yuan, Jiongru Liu, Qiu Meng, Jianhua Yin

**Affiliations:** College of Biotechnology and Bioengineering, Zhejiang University of Technology, Hangzhou 310014, China; 2111705001@zjut.edu.cn (Z.H.); 18757109635@163.com (Q.X.); 2111705006@zjut.edu.cn (M.Z.); yuannate@163.com (N.Y.); 17816106774@163.com (J.L.); mengq@zjut.edu.cn (Q.M.)

**Keywords:** quorum sensing, polycyclic aromatic hydrocarbons, biodegradation, cell surface hydrophobicity, *Sphingomonadales*

## Abstract

Members of the *Sphingomonadales* are renowned for their ability to degrade polycyclic aromatic hydrocarbons (PAHs). However, little is known about the regulatory mechanisms of the degradative pathway. Using cross-feeding bioassay, a functional LuxI/LuxR-type acyl-homoserine lactone (AHL)-mediated quorum sensing (QS) system was identified from *Croceicoccus naphthovorans* PQ-2, a member of the order *Sphingomonadales*. Inactivation of the QS system resulted in a significant decrease in PAHs degradation. The QS system positively controlled the expression of three PAH-degrading genes (*ahdA1e*, *xylE* and *xylG*) and a regulatory gene *ardR*, which are located on the large plasmid. Interestingly, the transcription levels of these three PAH-degrading genes were significantly down-regulated in the *ardR* mutant. In addition, bacterial cell surface hydrophobicity and cell morphology were altered in the QS-deficient mutant. Therefore, the QS system in strain PQ-2 positively regulates PAH degradation via two mechanisms: (i) by induction of PAH-degrading genes directly and/or indirectly; and (ii) by an increase of bacterial cell surface hydrophobicity. The findings of this study improve our understanding of how the QS system influences the degradation of PAHs, therefore facilitating the development of new strategies for the bioremediation of PAHs.

## 1. Introduction

Quorum sensing (QS) is a process of bacterial cell–cell communication that controls many important population-level behaviors, such as bioluminescence, biofilm formation, antibiotic resistance, and virulence factor production [1,2,3]. Bacteria produce and release signal molecules whose concentration accumulates as bacterial population density increases. When the signal molecules pass a specific threshold, QS alters global patterns of gene expression. In general, Gram-negative bacteria communicate using acyl-homoserine lactones (AHLs) as signal molecules [4]. AHLs are composed of a homoserine-lactone ring and a 4~18 carbon acyl chain that is occasionally modified by an oxo- or hydroxyl group at the 3-C position. The canonical AHL-mediated QS system was firstly discovered in *Vibrio fischeri* and then identified in many other Gram-negative bacteria. This system consists of two components, LuxI-type and LuxR-type proteins. The LuxI-type proteins are AHL synthases that catalyze the synthesis of AHLs, while LuxR-type proteins are transcription factors responsible for the perception of AHLs. LuxR-family proteins possess an N-terminal AHL-binding domain and a C-terminal DNA-binding domain. In the absence of AHLs, most LuxR proteins are unstable and fail to fold. However, the binding of AHLs to LuxR results in the stabilization and dimerization of LuxR. The LuxR-AHL complex binds to a conserved 20-bp palindrome termed “*lux* box” (5′-NNCT-N_12_-AGNN-3′) and then activates the expression of target genes [4,5].

Polycyclic aromatic hydrocarbons (PAHs) are a large class of hydrophobic organic compounds composed of two or more fused benzene rings arranged in various configurations, such as naphthalene, phenanthrene, anthracene, fluorine, and pyrene [6,7]. PAHs are widespread in the environment and most of them are persistent due to their poor aqueous solubility. Importantly, many PAHs are known to be toxic, mutagenic, and carcinogenic. Therefore, it is of great concern to develop efficient methods for removal of PAHs [8,9]. The conventional methods, which involve physical and chemical processes, have several drawbacks such as higher treatment cost and incomplete degradation of the pollutants. However, many of these drawbacks can be overcome by the use of bioremediation. It has been characterized that several microbial organisms can degrade PAHs via catabolism. It is especially interesting that the members of the order *Sphingomonadales* are regularly isolated from soils and marine sediments contaminated with PAHs. They are able to degrade various aromatic and/or xenobiotic compounds including PAHs [10,11,12]. Notably, members of the order *Sphingomonadales* contain glycosphingolipids rather than lipopolysaccharides in their outer membrane. This change may increase bacterial cell surface hydrophobicity and thus the degradation efficiency of hydrophobic PAHs [10].

Although the biochemical pathways for the degradation of PAHs have been widely studied for many years [9,12], little is known concerning the regulatory mechanism of the degradative pathway. Recently, a few studies have shown that the AHL-type QS system is involved in the regulation of PAHs degradation [13,14,15]. The QS system promotes aromatics degradation in *Pseudomonas aeruginosa* CGMCC1.860 but negatively affects phenanthrene removal in *Novosphingobium pentaromativorans* US6-1 [13,15]. Therefore, the mechanism underlying QS regulation on PAHs degradation is diverse and may vary among different species of bacteria. 

Several years ago, *Croceicoccus naphthovorans* PQ-2, a member of the family *Erythrobacteraceae* within the order *Sphingomonadales*, was isolated from marine biofilm [16,17]. The strain PQ-2 can degrade various PAHs and also produce AHLs [16]. However, the role of the AHL-type QS system in the degradation of PAHs remains unknown. In this study, we characterized the LuxI/LuxR-type QS system in *C. naphthovorans* PQ-2 and explored the involvement of QS regulation in the degradation of PAHs. We found that the QS system in strain PQ-2 positively regulates not only the transcription of PAH-degradative genes but also bacterial cell surface hydrophobicity.

## 2. Results

### 2.1. Identification of an AHL-Type QS System in C. naphthovorans PQ-2

*C. naphthovorans* PQ-2 can produce AHL molecules, but genes responsible for the AHL-type QS systemremain unidentified. According to the genome annotation information from NCBI, we found a pseudogene with a length of 515 bp (AB433_RS00085) that was annotated to encode an autoinducer synthase. This pseudogene contains only a partial coding region. In contrast, the full-length sequence of the gene (locus tag: Ga0111307_123190) can be obtained from the JGI database. This gene has a length of 657 bp and was designated *luxI*. Notably, a transposase gene (AB433_RS00065) and an integrase gene (AB433_RS00100) lie in the flanking sequence of the *luxI* gene.

**Figure 1:**
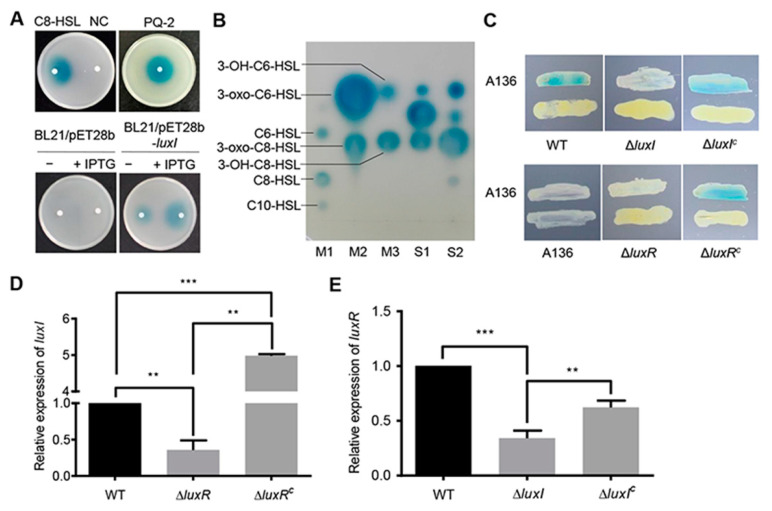
Identification of the LuxI/LuxR QS system. (**A**) Analysis of AHLs using the biosensor strain *A. tumefaciens* A136. AHLs were extracted from culture supernatants of *C. naphthovorans* PQ-2, *E. coli* BL21 harboring pET-28b(+) (BL21/pET28b) or pET-28b(+) expressing *luxI* (BL21/pET28b-*luxI*). C8-HSL and acidified ethyl acetate were used as positive control and negative control (NC), respectively. (**B**) Identification of AHLs by TLC. M1~M3, standard AHLs; S1, AHLs extracted from PQ-2; S2, AHLs extracted from *E. coli* BL21 expressing *luxI*. (**C**) Cross-feeding bioassay for detection of AHLs in *C. naphthovorans* strains. Δ*luxI*^C^ and Δ*luxR*^C^ represent the complemented version of Δ*luxI* and Δ*luxR*, respectively. (**D**,**E**) Relative expression levels of *luxI* (**D**) and *luxR* (**E**). The transcription level of the wild type was defined as 1.0. Experiments were performed in three biological replicates, and similar trends were observed. The representative data from three separate experiments are shown. Statistical significance of differences was analyzed by *t*-test; **, *p* < 0.01; ***, *p* < 0.001.

To determine whether the gene product of *luxI* is indeed an AHL synthase, the *luxI* gene was expressed in the *Escherichia coli* BL21 (DE3) strain that cannot produce any AHLs [18]. The *Agrobacterium tumefaciens* A136 was used as a biosensor strain to detect AHLs with medium and long acyl chains [19]. As expected, the culture extract of *E. coli* harboring the empty plasmid did not affect the biosensor strain. However, the recombinant *E. coli* sample induced a blue coloration on the indicator plate, which results from the expression of *β*-galactosidase in *A. tumefaciens* A136 (Figure 1A). TLC analysis showed that the strain PQ-2 produces three AHLs, C6-HSL and C8-HSL with an oxo- or hydroxyl group at the 3-C position, and an unknown AHL that needs to be further investigated (Figure 1B). Similar results were also observed in the crude AHLs extract from the recombinant *E. coli* cells (Figure 1B).

To further confirm whether the *luxI* gene is active in *C. naphthovorans* PQ-2, an *rpsL*-based markerless gene deletion system for Sphingomonads was employed to construct the ∆*luxI* strain [20]. Cross-feeding bioassay results showed that the ∆*luxI* strain failed to induce a blue coloration of *A. tumefaciens* A136, while expression of the *luxI* gene under its native promoter regained the ability to generate a blue coloration (Figure 1C). Therefore, the *luxI* gene is responsible for the synthesis of AHLs in *C. naphthovorans* PQ-2. 

Given that the expression of *luxI* depends on a LuxR-type autoinducer-responsive regulator, the cognate gene for LuxR was identified from the PQ-2 genome. The locus tag Ga0111307_123182, which locates 6.3 kb upstream of the *luxI* gene, was predicted to encode a LuxR homolog. Accordingly, we renamed this gene *luxR*. Similar to the strain lacking *luxI*, the ∆*luxR* strain no longer induced a blue coloration of the biosensor strain, which could be restored by genetic complementation (Figure 1C). In addition, the transcription of *luxI* was significantly reduced in the ∆*luxR* strain (Figure 1D), while the transcription of *luxR* was down-regulated in the ∆*luxI* strain (Figure 1E). Both transcription levels were restored by complementation (Figure 1D,E). Collectively, we can conclude that *C. naphthovorans* PQ-2 possesses a functional AHL-type QS system, which is composed of an AHL synthase LuxI and an autoinducer-responsive regulator LuxR.

### 2.2. The AHL-Type QS System Is Crucial for the Degradation of PAHs

**Figure 2:**
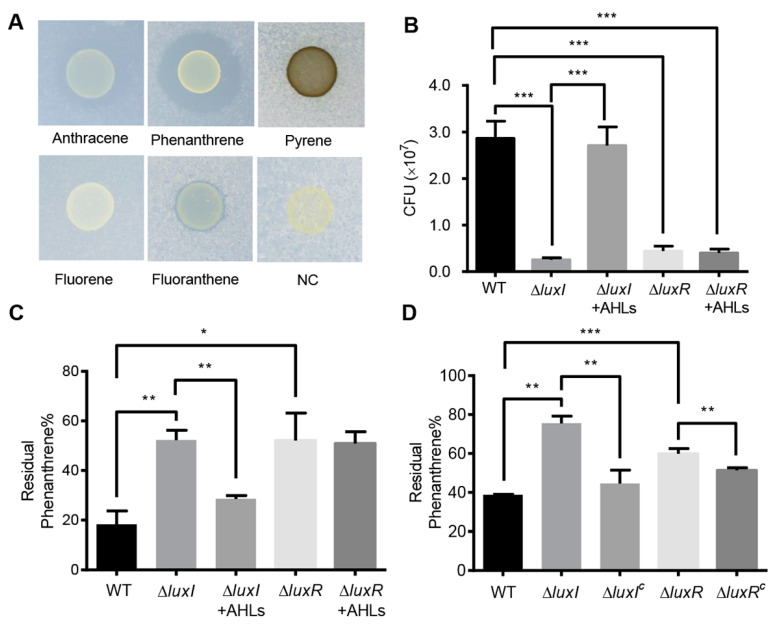
Effects of the QS system on phenanthrene degradation. (**A**) The degradation of PAHs by *C. naphthovorans* PQ-2. (**B**) Growth of *C. naphthovorans* strains in minimal medium containing phenanthrene as the sole carbon and energy source. Colony forming units (CFUs) were determined for each sample. (**C**,**D**) Percentage of residual phenanthrene in the medium after incubation for 72 h. Experiments were performed in three biological replicates, and similar trends were observed. The representative data from three separate experiments are shown. Statistical significance of differences was analyzed by *t*-test: *, *p* < 0.05; **, *p* < 0.01; ***, *p* < 0.001.

To investigate the degradation of PAHs, *C. naphthovorans* PQ-2 was dropped onto minimal medium plates containing different PAHs (fluorene, phenanthrene, anthracene, fluoranthene, and pyrene) as the sole carbon and energy source. As shown by the results in Figure 2A, growth of PQ-2 was observed on all the plates tested, suggesting that this bacterium can use various PAHs as the sole carbon and energy source. Notably, PQ-2 had the highest degradation ability for phenanthrene and anthracene, which forms a halo around bacterial colonies. Phenanthrene was then used in subsequent studies. 

To explore the involvement of the AHL-type QS system in the degradation of PAHs, growth of the ∆*luxI* and ∆*luxR* strains was measured in liquid culture media. Deletion of *luxI* and *luxR* per se did not affect bacterial growth in nutrient-rich P5Y3 medium (Figure S1). However, in minimal medium with phenanthrene as the sole carbon and energy source, the colony-forming units (CFUs) of strains lacking either *luxI* or *luxR* were significantly reduced when compared to the wild type (Figure 2B). CFUs of the ∆*luxI* strain were restored by exogenous addition of AHLs, which were extracted from the wild type of PQ-2. On the contrary, the addition of AHLs was unable to restore the CFUs of the Δ*luxR* strain. The residual phenanthrene in the supernatants was analyzed by HPLC. Both ∆*luxI* and ∆*luxR* strains displayed an obvious reduction in phenanthrene degradation (Figure 2C,D). The degradation ability of the ∆*luxI* strain but not the Δ*luxR* strain was almost reversed to the wild type when supplemented with the AHLs extracted from the PQ-2 (Figure 2C). Moreover, the expression of either *luxI* or *luxR* in the corresponding mutants partially recovered the ability of phenanthrene degradation (Figure 2D). These results collectively indicate that the AHL-mediated QS system plays an important role in the degradation of phenanthrene in *C. naphthovorans* PQ-2.

### 2.3. PQ-2 Degrades Phenanthrene through the Salicylic Acid Pathway

The common microbial pathway for PAHs degradation involves the formation of catechol, which will then be transformed into the tricarboxylic acid (TCA) cycle [8]. To determine the degradation pathway of phenanthrene in *C. naphthovorans* PQ-2, the degradation products were analyzed by GC-MS (Figure S2). After degradation by strain PQ-2 for 48 h, the samples were separated into neutral and acidic fractions. Phenanthrene was detected in the neutral fraction, which results from the incomplete degradation. By contrast, the acidic metabolites of phenanthrene contain salicylic acid and catechol, suggesting that the strain PQ-2 also transforms phenanthrene to the common intermediate catechol.

### 2.4. The Large Plasmid Is Responsible for the Degradation of Phenanthrene

**Figure 3:**
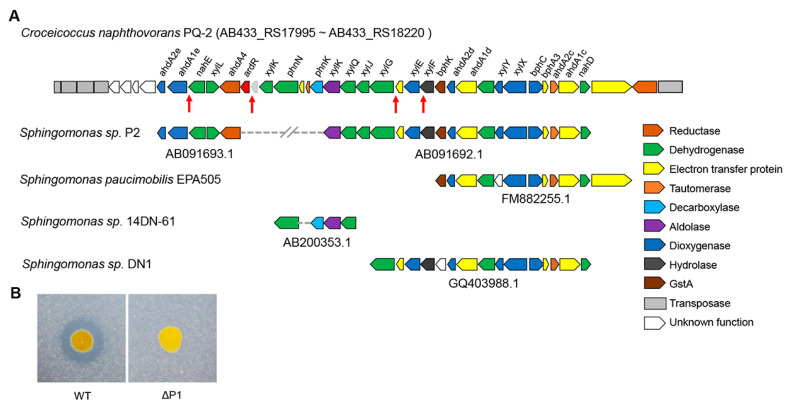
The large plasmid P1 is essential for phenanthrene degradation. (**A**) Analysis of the PAH-degrading gene clusters in *C. naphthovorans* PQ-2. The red arrows show the “*lux* box” site in the promoter region of several PAH-degrading genes. (**B**) Effect of plasmid P1 removal on phenanthrene degradation.

The complete genome of *C. naphthovorans* PQ-2 is composed of a chromosome (3.54 Mb) and two plasmids, P1 (0.19 Mb) and P2 (0.13 Mb). Sequence analysis demonstrated that the large plasmid P1 possesses a putative PAH-degrading cluster (from AB433_RS17995 to AB433_RS18220), which shares high identities with several aromatic compound degradation gene clusters in other *Sphingomonas* strains (Figure 3A). For example, the nucleotide sequence from AB433_RS18095 to AB433_RS18120 of PQ-2 has 95% identity with the *phnIJKN* cluster of *Sphingomonas* sp. 14DN-61, which has been confirmed to be involved in PAHs degradation [21]. Similar to the aforementioned *luxI* gene, several transposase genes are located in the flanking sequence of the putative PAH-degrading cluster.

To explore whether the large plasmid P1 is involved in phenanthrene degradation, PQ-2 was treated with rifampicin to eliminate the plasmid. The strain lacking the P1 (ΔP1) was screened in the presence of 20 μg/mL rifampicin. Compared to the wild type, the ΔP1 strain was no longer able to degrade phenanthrene (Figure 3B), indicating that the plasmid P1 is required for phenanthrene degradation.

### 2.5. The AHL-Type QS System Regulates the Expression of PAH-Degrading Genes

**Figure 4:**
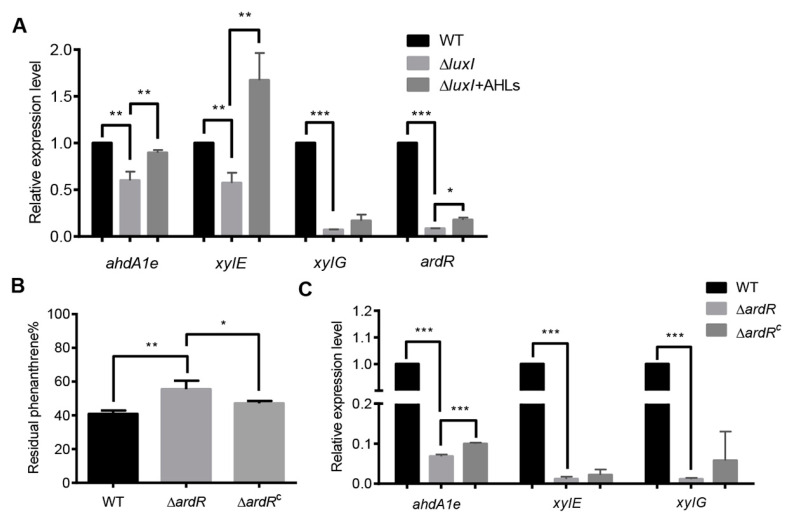
The QS system regulates the expression of PAH-degrading genes. (**A**) The relative transcriptional levels of PAH-degrading genes in the Δ*luxI* strain with or without exogenous AHLs. (**B**) Percentage of residual phenanthrene after three days degradation by the Δ*ardR* and its complemented version (Δ*ardR*^C^). (**C**) The relative transcriptional levels of PAH-degrading genes in the Δ*ardR*. Experiments were performed in three biological replicates, and similar trends were observed. The representative data from three separate experiments are shown. Statistical significance of differences was analyzed by *t*-test: *, *p* < 0.05; **, *p* < 0.01; ***, *p* < 0.001.

To investigate how the AHL-mediated QS system regulates phenanthrene degradation in *C. naphthovorans* PQ-2, we analyzed the transcription levels of genes within the PAH-degrading gene cluster in the Δ*luxI* strain. Based on gene annotation and the degradative pathway of phenanthrene in PQ-2, eight PAH-degrading genes within the cluster were chosen for qRT-PCR analysis, including *ahdA2e*, *ahdA1e*, *bphC*, *xylG*, *xylE*, *xylX*, *xylY* and *ahdA2c*. Among them, the transcription levels of three PAH-degrading genes (*ahdA1e*, *xylE* and *xylG*) were significantly down-regulated in the Δ*luxI* strain, which could be fully (*ahdA1e* and *xylE*) or partially (*xylG*) rescued when supplemented with exogenous AHLs extracted from PQ-2 broth (Figure 4A). The *ahdA1e* gene encodes the *α* subunit of the aromatic ring-hydroxylating dioxygenase, while the *xylE* and *xylG* genes encode a catechol 2, 3 dioxygenase (C23O) and a 2-hydroxymuconic semialdehyde dehydrogenase, respectively. Notably, the promoter region of all three PAH-degrading genes possess the conserved “*lux* box” element.

We also determined the expression of AB433_RS18080 gene (now designated as *ardR* for aromatic degradation regulator), which is predicted to encode a sigma-54-dependent Fis family transcriptional regulator. As shown by the results in Figure 4A, the expression of *ardR* was dramatically reduced in the absence of *luxI*, which could be partially restored when exogenous addition of the PQ-2 AHLs extract. These results indicate that ArdR may be involved in the regulation of PAHs degradation. To confirm, the *ardR* gene was deleted from the plasmid P1 and then the resultant mutant (Δ*ardR*) was subjected to phenanthrene degradation assay (Figure 4B). Deletion of the *ardR* gene led to a significant decrease in the degradation of phenanthrene, which could be relieved by the expression of the *ardR* gene *in trans*. The upstream sequence of *ardR* also contains a conserved “*lux* box” sequence. Interestingly, the transcription levels of these three PAH-degrading genes were sharply reduced in the Δ*ardR* strain when compared to the wild type (Figure 4C). Relative expression of *ahdA1e* but not *xylE* and *xylG* was partially restored in the complemented version of *ardR* (Δ*ardR*^C^) *in trans*.

### 2.6. The AHL-Type QS System Affects Bacterial Cell Surface Hydrophobicity

**Figure 5:**
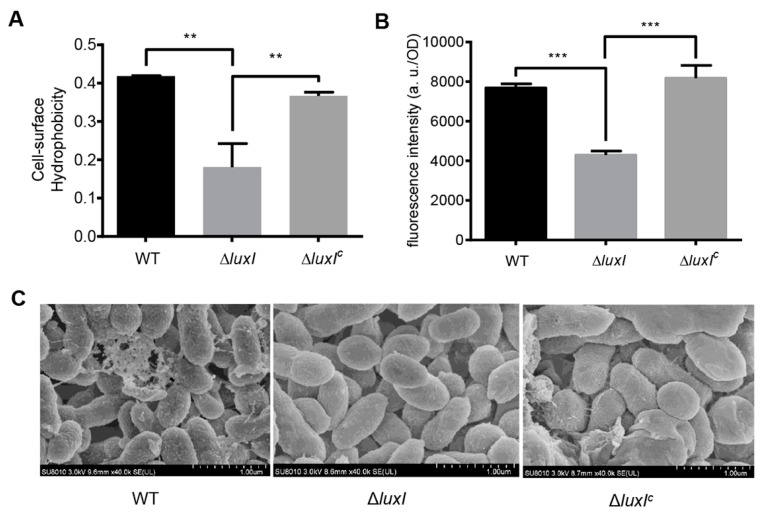
The QS system regulates bacterial cell surface properties. (**A**) Cell surface hydrophobicity of the Δ*luxI* and its complemented version (Δ*luxI*^C^). (**B**) Bis-ANS fluorescence intensity. (**C**) Scanning electron micrographs. For (**A**,**B**), experiments were performed in three biological replicates, and similar trends were observed. The representative data from three separate experiments are shown. Statistical significance of differences was analyzed by *t*-test: **, *p* < 0.01; ***, *p* < 0.001.

Bacterial cell surface hydrophobicity is crucial for the degradation of organic pollutants in the environment [15,22]. To determine whether the AHL-type QS system affects cell surface properties in *C. naphthovorans* PQ-2, microbial adherence to hydrocarbons (MATH) assay was performed to determine cell surface hydrophobicity (Figure 5A). Compared to the wild type, deletion of the *luxI* gene led to a ~2-fold decrease in the cell surface hydrophobicity, which could be recovered by expression of the *luxI* gene *in trans*. To further investigate this, we measured the content of glycosphingolipids by Enzyme-linked immunosorbent assay (ELISA), but no significant change was observed in all tested strains (Figure S3). We next determined the content of hydrophobic proteins on the cell surface using the fluorescent probe bis-ANS (Figure 5B). The Δ*luxI* strain displayed a significant decrease in the bis-ANS fluorescence when compared to the wild type, while expression of the *luxI* gene restored the fluorescence of the complemented strain to the level of the wild type. Therefore, the Δ*luxI* strain has lower amount of hydrophobic proteins than the wild type on the cell surface.

Cell morphology was also observed by scanning electron microscopy (SEM) (Figure 5C). Results showed that the cell surface morphology of the Δ*luxI* strain was quite different from that of the wild type. The wild type strain exhibited a rough cell surface with many extracellular matrix components, whereas the Δ*luxI* strain had a smooth cell surface without extracellular matrix components.

## 3. Discussion

**Figure 6:**
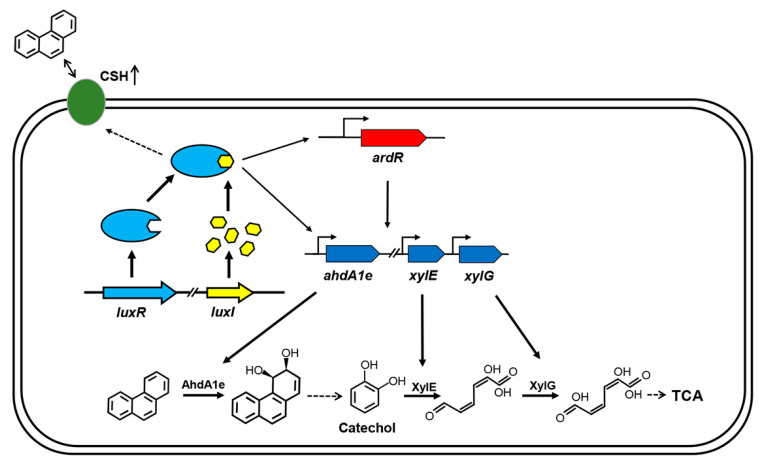
Model for QS regulation on phenanthrene degradation in *C. naphthovorans* PQ-2. The QS system positively regulates PAH degradation via multiple mechanisms. The LuxR-AHL complex may bind to the “*lux* box” and then directly regulate the transcriptional levels of three PAH-degrading genes, including *ahdA1e*, *xylE* and *xylG*. The LuxR-AHL complex may also indirectly modulate the expression of these PAH-degrading genes, which is mediated by the transcriptional regulator ArdR. Moreover, the QS system enhances bacterial cell surface hydrophobicity (CSH), thus affecting the attachment of cells to PAHs. Similar to other AHL-type QS systems, the LuxR-AHL complex in PQ-2 has a positive feedback effect on the expression of *luxR* and *luxI*. For clarity, the secretion and uptake of AHLs are omitted in the model.

The biochemical pathways for the degradation of PAHs by microorganisms have been extensively studied in the last few decades [9,12]. In comparison, little is known about the regulatory mechanisms of the degradative pathways. Recently, the AHL-type QS system was reported to be involved in the regulation of PAHs degradation in some Gram-negative bacteria. The QS system positively controls the degradation of PAHs in *P. aeruginosa* strains through either biofilm formation or induction of key degradative genes [13,14,23]. In this study, we characterized an AHL-type QS system from *C. naphthovorans* PQ-2, a member of the order *Sphingomonadales.* This QS system positively regulates PAHs degradation not only by the induction of PAH-degrading genes but also by an increase of cell surface hydrophobicity (Figure 6).

The *Sphingomonadaceae*, the largest family within the order *Sphingomonadales*, are renowned for their ability to degrade recalcitrant compounds and xenobiotics including PAHs [10,24]. *C. naphthovorans* PQ-2 belongs to the family *Erythrobacteraceae*, which is the second largest family within the order *Sphingomonadales* [17]. Our results demonstrate that *C. naphthovorans* PQ-2 can degrade PAHs containing three and four rings, such as phenanthrene, anthracene, fluorene, fluoranthene and pyrene. Among them, PQ-2 has the highest degradation ability for three-ring molecules phenanthrene and anthracene. These two PAHs are known to be more susceptible to biodegradation, since they are more volatile and more soluble in water [25]. It is unclear whether PQ-2 is capable of using PAHs with five or more than five rings.

The genes involved in PAHs degradation in the *Sphingomonadaceae* are very often located on large plasmids (megaplasmids) [10,23,26]. Consistently, our results show that the large plasmid P1 in *C. naphthovorans* PQ-2 is responsible for the degradation of phenanthrene. This megaplasmid possesses a PAH-degrading gene cluster which is composed of 32 open reading frames. There are several transposase genes located in the flanking regions of the gene cluster, implying that this cluster may be transferred horizontally from other bacteria. The degradative pathway for phenanthrene in *C. naphthovorans* PQ-2 is also in agreement with that in the members of the family *Sphingomonadaceae* [8,10,27]. Under aerobic conditions, the aromatic ring-hydroxylating dioxygenase (including the *α* subunit AhdA1e and *β* subunit AhdAle2) catalyzes the initial reaction in the degradative pathway, resulting in the modification of the aromatic ring. After several steps, *C. naphthovorans* PQ-2 degrades phenanthrene to the common intermediate catechol. Subsequently, the catechol 2, 3 dioxygenase XylE and 2-hydroxymuconic semialdehyde dehydrogenase XylG convert catechol to 2-hydroxymuconate, which will be further transformed to TCA cycle (Figure 6).

Although the research on regulation of the PAH-degrading pathways is limited, the existing studies demonstrated that the AHL-type QS system is involved in the regulation of PAHs degradation process in *P. aeruginosa* CGMCC1.860 [13]. The AHL-type QS system in this bacterium directly and indirectly controls the expression of catechol 2,3-dioxygenase gene, resulting in the enhanced aromatics biodegradation [13]. Similar results were observed in our studies on *C. naphthovorans* PQ-2. The AHL-type QS system in *C. naphthovorans* PQ-2 positively regulates the expression of three PAH-degrading genes (including *ahdA1e*, *xylE* and *xylG*), as well as the regulatory gene *ardR*. It is worth mention that the upstream regions of these genes contain a conserved “*lux* box” sequence (5′-NNCT-N_12_-AGNN-3′). Therefore, the LuxR-AHL complex may bind to the “*lux* box” site and then directly regulate the expression of these target genes, thus affecting phenanthrene degradation (Figure 6). 

The transcription of the three PAH-degradative genes is also regulated by the *ardR* gene, which is located within the PAH-degrading gene cluster. The transcriptional regulators for PAHs degradation have been reported in several *Pseudomonas* and *Novosphingobium* species [13,28,29]. It is also possible that the AHL-type QS system in *C. naphthovorans* PQ-2 indirectly modulates the expression of PAH-degrading genes, which is mediated by the transcriptional regulator ArdR (Figure 6). The expression of PAH-degrading genes may be regulated in a hierarchical manner. The AHL-mediated QS system is at the apex of the regulatory cascade, while the specific transcriptional regulator sets up the second cascade driving the expression of PAH-degrading genes.

In addition to up-regulation of the degradative enzymes, our results show that the AHL-type QS system in *C. naphthovorans* PQ-2 also positively controls bacterial cell surface hydrophobicity. Many studies have demonstrated that there is a positive correlation between cell surface hydrophobicity and PAHs degradation [15,22,30,31]. PAH-degrading bacteria normally have high cell surface hydrophobicity, which stimulates the direct attachment of bacterial cells to hydrophobic surface including PAHs and the partition of dissolved PAHs to the cell surface [22]. As a result, PAHs enter into bacterial cell for biodegradation. Thus, the QS system of *C. naphthovorans* PQ-2 facilitates the attachment of bacterial cells to PAHs and then enhances the uptake of PAHs. Once PAHs enter into the cytoplasm of the bacterial cell, they will induce the expression of PAH-degrading genes and then the degradation of PAHs (Figure 6). Due to the presence of glycosphingolipids, the cell surface of sphingomonads is more hydrophobic than those of other bacteria [10]. However, the enhanced cell surface hydrophobicity in *C. naphthovorans* PQ-2 is not derived from glycosphingolipids, but related to hydrophobic proteins on the cell surface. There are many factors that can affect cell surface hydrophobicity. For example, it has been reported that biosurfactants secreted by *Bacillus subtilis* and *P. aeruginosa* enhance bacterial cell surface hydrophobicity, resulting in higher uptake and use of pyrene [22,32]. The detailed mechanism in *C. naphthovorans* PQ-2 needs to be further investigated. 

In contrast to our results, a recent study demonstrated that the AHL-type QS system negatively regulates phenanthrene removal in *N. pentaromativorans* US6-1, a member belongs to the family *Sphingomonadaceae* [15]. Deletion of the AHL-type QS system in US6-1 increases phenanthrene removal efficiency. This phenomenon is also related to PAH-degrading genes, since the relative expression levels of 12 PAH-degrading genes are up-regulated in QS-deficient mutants. Interestingly, QS-deficient mutants have significantly higher cell surface hydrophobicity, which partially results from the increase of hydrophobic glycosphingolipids. Therefore, the two AHL-type QS systems play an opposite role in the regulation of PAHs degradation in *N. pentaromativorans* US6-1 and *C. naphthovorans* PQ-2. At present, the underlying mechanism remains unclear. It should be noted that *N. pentaromativorans* US6-1 was isolated from muddy sediment [33], while *C. naphthovorans* PQ-2 was isolated from marine biofilm [17]. Biofilm-based bioremediation is one of the most efficient approaches for the decontamination of pollutants [14,34]. Thus, *C. naphthovorans* PQ-2 has a great potential in bioremediation of PAHs.

## 4. Materials and Methods

### 4.1. Bacterial Strains, Plasmids, Primers and Culture Conditions

The bacterial strains and plasmids used in this study are listed in Table 1. The primers used in this study are listed in Table S1. *E. coli* and *A. tumefaciens* A136 (pCF218/pCF372) were grown in Luria-Bertani (LB) medium at 37 °C and 30 °C, respectively. For cross-feeding bioassay, *C. naphthovorans* PQ-2 was cultured in P5Y3 medium at 30 °C [17]. For biodegradation assay, *C*. *naphthovorans* was cultivated at 30 °C in marine minimal medium supplemented with phenanthrene (200 mg/L) [17]. When needed, the medium was supplemented with chemicals at the following concentrations: ampicillin (Amp), 100 μg/mL; gentamycin (Gm), 50 μg/mL; kanamycin (Km), 50 μg/mL; streptomycin (Sm), 100 μg/mL; 2,6-diaminopimelic acid (DAP), 0.3 mmol/L.

### 4.2. Expression of AHL Synthase of Strain PQ-2 in E. coli

The full-length sequence of *luxI* encoding AHL synthase was obtained from the JGI database (locus tag: Ga0111307_123190). PCR amplification was performed with the primers containing restriction enzyme sites (Table S1). The resulting PCR product was cloned into the expression vector pET-28b(+), and then the recombinant plasmid pET-28b(+)-*luxI* was transformed into *E. coli* BL21 (DE3). Finally, the recombinant vector was verified by sequencing.

### 4.3. Extraction and Analysis of AHLs

The AHLs produced by *E. coli* and *C. naphthovorans* were extracted from culture supernatants as previously described [36,37]. 100 mL of overnight bacterial culture was centrifuged at 8000 rpm for 10 min. The supernatant was extracted three times with an equal volume of acidified ethyl acetate. The combined extract from the organic phase was evaporated on a rotatory evaporator at 50 °C. The dried residue was dissolved into 1 mL of acidified ethyl acetate and stored at −20 °C. 

The profile of AHLs was determined using thin layer chromatography (TLC) [36]. Briefly, the separation of AHL extracts was carried out on a TLC plate (TLC aluminum sheets, 20 cm × 20 cm, Silica gel 60 F254; Merck, Germany) using methanol–ultrapure water (60:40, *v*/*v*) as mobile phase. After chromatography and air-dry, the plate was covered by LB agar containing the reporter strain *A. tumefaciens* A136, which is capable of sensing medium-/long-chain AHLs [19]. X-gal was then spread onto the LB agar. The profile of AHLs was analyzed after incubation for 24 h at 30 °C. All standard AHLs including C6-HSL, C8-HSL, C10-HSL, 3-OH-C6-HSL, 3-oxo-C6-HSL, 3-OH-C8-HSL, and 3-oxo-C8-HSL were purchased from Sigma (Bejing, China).

### 4.4. Cross-Feeding Bioassay

The AHLs produced by bacteria were measured by cross-feeding bioassay as described elsewhere [16,36]. The tested bacteria and the biosensor *A. tumefaciens* A136 were streaked in parallel on LB-P5Y3 plate (containing equal amounts of LB and P5Y3) supplemented with X-gal (40 μg/mL). The cross-feeding plate was then cultivated at 30 °C for 12 h before color visualization. 

For analysis of AHL extracts, 2 μL of each AHL extract was dropped onto a sterile filter paper on the indicator plate. The plate was incubated for 12 h at 30 °C and then photographed. C8-HSL and acidified ethyl acetate were used as positive control and negative control, respectively.

### 4.5. Markerless Gene Deletion Mutagenesis and Complementation

The gene deletion mutant of *C. naphthovorans* PQ-2 was constructed by an *rpsL*-based markerless gene deletion system [20] with a little modification. In brief, two fragments flanking the target gene were amplified by PCR with primers containing the restriction enzyme sites and then were joined by fusion PCR (Table S1). The fusion fragment and the suicide plasmid pAK405 were ligated together by T4 DNA ligase and transformed into *E. coli* WM3064 (DAP auxotroph) [35]. The resulting plasmid was transferred into *C. naphthovorans* PQ-2 via conjugation. Integration of the mutagenesis constructs into the chromosome was selected by resistance to kanamycin and verified by PCR. The correct transconjugant was grown in P5Y3 broth for three days and then plated onto the P5Y3 plate supplemented with streptomycin. Kanamycin-sensitive and streptomycin-resistant colonies were screened by PCR for deletion of the targeted gene. Finally, the deletion mutations were verified by sequencing.

The broad-host-range plasmid pBBR1MCS-5 was used for the genetic complementation of mutants [36]. A fragment containing the gene of interest and its native promoter was amplified by PCR and then cloned into pBBR1MCS-5. The resulting recombinant plasmid was then transferred into the corresponding mutant via conjugation.

### 4.6. RNA Isolation and Quantitative Real-Time PCR (qRT-PCR)

Total RNA from *C. naphthovorans* strains was isolated using the RNAiso Plus Kit (TaKaRa, Dalian, China) according to the manufacturer’s instructions. The qRT-PCR analysis was performed with a CFX Connect Real-Time PCR Detection System (BioRad, Hercules, CA, USA) as described previously [36]. The cycle threshold (*C_T_*) values for each gene of interest were normalized against the *C_T_* values of the 16S rRNA gene. The relative expression level of each gene of interest was determined from three replicates in a single experiment.

### 4.7. Analysis of Phenanthrene and Its Metabolites

For phenanthrene analysis, the sample was extracted with ethyl acetate and dried under vacuum. Then it was dissolved in acetonitrile, filtrated, and subjected to HPLC analysis [38]. The column for the measurement was InertsilODS-3 C18 column (4.6 × 250 mm, 5 μm), and the mobile phase was methanol/water (90:10) at a flow rate of 1.0 mL/min using a Waters 2487 dual-wavelength detector. 

The phenanthrene metabolites were analyzed by gas chromatography–mass spectrometry (GC-MS), as previously described [39,40]. For brief, phenanthrene was allowed to degrade for 48 h. The culture was centrifuged, and the supernatant was then acidified to pH 2.3 with HCl and extracted with ethyl acetate. The organic phase was extracted three times with an equal volume of NaOH (10 mmol/L). The remaining organic phase was dried over anhydrous sodium sulfated and concentrated to 5mLof ethyl acetate (neutral fraction). The aqueous phase was acidified to pH 2.3 with HCl and extracted with ethyl acetate (acidic fraction). The GC-MS analysis of neutral and acidic fractions was carried out with/without derivatization. 

### 4.8. Microbial Adhesion to Hydrocarbons (MATH) Test

The MATH test was carried out to determine bacterial cell surface hydrophobicity as described previously [15,41] with some modifications. After incubation, bacterial cultures were resuspended in phosphate-buffered saline (PBS) and the OD_600_ values were measured (OD_0_). Then, 4 mL of xylene was added to 8 mL of the suspension. The mixtures were vortexed for 15 s, and allowed phase separation for 20 min at room temperature. Subsequently, the OD_600_ of the aqueous phase of the suspension was measured (OD_1_). The cell surface hydrophobicity values were calculated according to the following equation:

Cell surface hydrophobicity (%) = [(OD_0_ − OD_1_)/OD_0_] × 100%. (1)

### 4.9. Determination of Hydrophobic Proteins Using Fluorescent Dye

The fluorescent probe bis (8-anilinonaphthalene-1-sulfonate) (bis-ANS) was used to determine hydrophobic proteins [42]. Bacterial cultures were resuspended in PBS (pH = 7.4) and the bacterial cell density was adjusted to 0.15 at OD_600_. 30 µL bis-ANS was added to 170 µL of these samples to achieve a final concentration of 5 µmol/L. The fluorescence intensity of bis-ANS was measured by the UV-Vis spectrophotometer (Molecular Devices SpectraMax M4, Sunnyvale, CA, USA). The excitation and emission wavelengths were 385 nm and 530 nm, respectively.

### 4.10. Scanning Electron Microscopy (SEM)

*C. naphthovorans* strains were cultivated in marine minimal medium supplemented with phenanthrene. 1 mL of bacterial culture was centrifuged at 4000 rpm for 5 min. The pellet was fixed with 4% (*v*/*v*) glutardialdehyde overnight at 4 °C, washed with PBS and dehydrated by passage through a graded ethanol series (30%, 50%, 70%, 80%, 90%, and 100%, *v*/*v*), subsequently dehydrated with liquid carbon dioxide and coated with platinum powder. The bacterial cell morphology was observed with SEM (Hitachi SU8010, Tokyo,Japan) [43].

## 5. Conclusions

Sphingomonads are biotechnologically interesting organisms for their potential in the bioremediation of PAHs. This study indicates that the AHL-mediated QS system in *C. naphthovorans* PQ-2 positively regulates the degradation of PAHs via two mechanisms: (i) by induction of PAH-degrading genes expression directly and/or indirectly and (ii) by an increase of cell surface hydrophobicity. The findings of this study improve our understanding of the involvement of QS regulation in the degradation of PAHs in Sphingomonads, therefore facilitating the development of new strategies for the bioremediation of PAHs.

## Figures and Tables

**Table 1:** Strains and plasmids used in this study.

Strain or Plasmid	Description	Source or Reference
Strains		
*C. naphthovorans* PQ-2	Wild type	[16]
*A. tumefaciens* A136 (pCF218/pCF372)	biosensor strain for medium-/long-chain AHLs	[19]
*E. coli* DH5α	Host strain for cloning	Lab stock
*E. coli* BL21(DE3)	Expression host for pET28b(+)	TransGen Biotech
*E. coli* WM3064	Donor strain for conjugation	[35]
Δ*luxI*	Mutant of strain PQ-2 with deletion of *luxI*	This study
Δ*luxR*	Mutant of strain PQ-2 with deletion of *luxR*	This study
Δ*ardR*	Mutant of strain PQ-2 with deletion of *ardR*	This study
ΔP1	Mutant of strain PQ-2 with deletion of the large plasmid	This study
Δ*luxI*^C^	Complemented strain of Δ*luxI*	This study
Δ*luxR*^C^	Complemented strain of Δ*luxR*	This study
Δ*ardR*^C^	Complemented strain of Δ*ardR*	This study
Plasmids		
pBBR1MCS-5	Gm^r^, broad-host vector	[36]
pET-28b(+)	T7 expression vector	Novagen
pET-28b(+)-*luxI*	pET-28b(+) containing AHL synthase LuxI	This study
pAK405	Km^r^, sphingomonad suicide vector	[20]

## Supplementary Materials

Supplementary Materials can be found at https://www.mdpi.com/1422-0067/21/15/5548/s1.

## Abbreviations

QS	Quorum Sensing
AHL	Acylated Homoserine Lactone
PAHs	Polycyclic Aromatic Hydrocarbons
CSH	Cell Surface Hydrophobicity
DAP	2,6-Daminopimelic Acid
MATH	Microbial Adhesion to Hydrocarbons
CFUs	Colony-forming Units
OD	Optical Denstity
TCA	Tricarboxylic Acid
SEM	Scanning Electron Microscopy
TLC	Thin Layer Chromatography
ArdR	Aromatic Degradation Regulator
