# Supplementary material for: The LuxI/LuxR-Type Quorum Sensing System Regulates Degradation of Polycyclic Aromatic Hydrocarbons via Two Mechanisms
Source: Int J Mol Sci. 2020 Aug 3;21(15):5548. doi: 10.3390/ijms21155548 (PMC7432010; doi:10.3390/ijms21155548)
Supplement: Supplementary file 1 [file ijms-21-05548-s001.pdf]

Table S1. Primers used in this study.

Primer	Sequence (5' to 3')
In-frame deletion	
luxI-FO	TTCACCGAACGCTTCATTT
luxI-RO	GCCGAGACTGTAGCCGATT
luxI-5O	CGCGGATCCGTTCTTGATGCGATGGCTGAG
luxI-5I	TGTTTAAACTTAGTGGATGGGGACTGCTCGCATCTCATC
luxI-3I	CCCATCCACTAAGTTTAAACACTGGCAGCATGACTGCGC
luxI-3O	CGCAAGCTTGGACATCCTCGGTCTGGTTT
luxR-FO	AGCGGTAGCGGCGGAGTCAA
luxR-RO	CAAGGTAGCTGCGTCCAATAT
luxR-5O	CGCGGATCCCCAAACGAGCGAGCAATG
luxR-5I	TGTTTAAACTTAGTGGATGGGATATGCGTACATAGACCT
luxR-3I	CCCATCCACTAAGTTTAAACAGACGTCGGATAGACGCAC
luxR-3O	CGCAAGCTTCTCAACGGCAATCGGACA
ardR-FO	AAACCTGAAGCCGGTCACG
ardR -RO	GTTCAGCGGCACCAAGACT
ardR -5O	TGCTCTAGA GCAGTTGAAATGGGTCGCTA
ardR -5I	TGTTTAAACTTAGTGGATGGG GTGCCTCATCATCCATCACT
ardR -3I	CCCATCCACTAAGTTTAAACA TGTAGAGCGTTCGGCCATT
ardR -3O	CGCAAGCTTAGGTCTCTACGCGCTTTTGG
Complementation	
luxI -Fc	CCCTCGAGGACCATACCATCTGCGGCAT
luxI - Rc	CGGGATCCGAAGGAGGCTTCCAAACCGT
luxR-Fc	CCCTCGAGCCACCTATCAGTCCAGACGC
luxR - Rc	CGGGATCCTGGCTCGATGGTGTGATTGT
ardR -Fc	TGCTCTAGACCAGTACACCTCGGTGATCG
ardR - Rc	CGCAAGCTT CCGCGGTGACTGTCTATCTT
qRT-PCR	
ahdA1e-qrt-F	ATGTCTTCGGCAACCTACGTGAAC
ahdA1e-qrt-R	CGGTAGGATGTCTCGCCAATGAAG
xylE-qrt-F	GTGACCGAGGAATTGCTGGATGAG
xylE-qrt-R	CGAGGAAGAACGACGTGTGATGG
XylG-qrt-F	ACAACGTGGTGCATGGTTTC
XylG-qrt-R	TACCGAACGCGACAATCCTT
ArdR-qrt-F	CGATGGTTGAACGTGCGATT
ArdR-qrt-R	CAACAGTCCGACCAAGTGAC
luxI-qrt-F	AATGGTGCAAACCTGGTCGAT
luxI-qrt-R	CTCGCGAAATGCCTCATCCT
luxR-qrt-F	CTTTGAGCTGTGCACTGGGA
luxR-qrt-R	ATGACGGTTTCGGTGCTGAT
Heterologous expression	
PQ2-luxI-F	CGCGAATTCTATGCGAGCAGTCATCAAGTT
PQ2-luxI-R	CGCAAGCTTTGCTGCCAGCTCCATACCAC

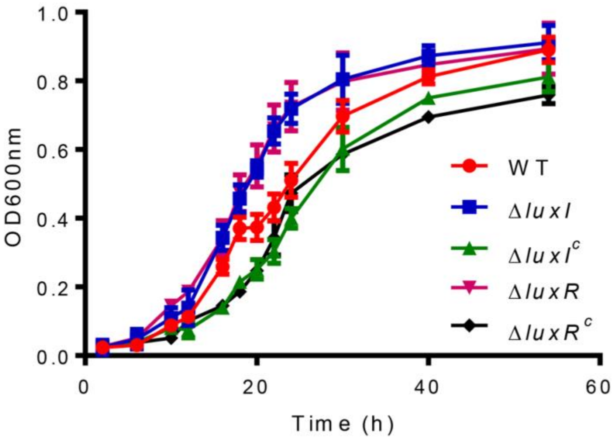

Figure S1. Effects of the QS system on growth rate of *C. naphthovorans* PQ-2 in nutrient-rich P5Y3 medium

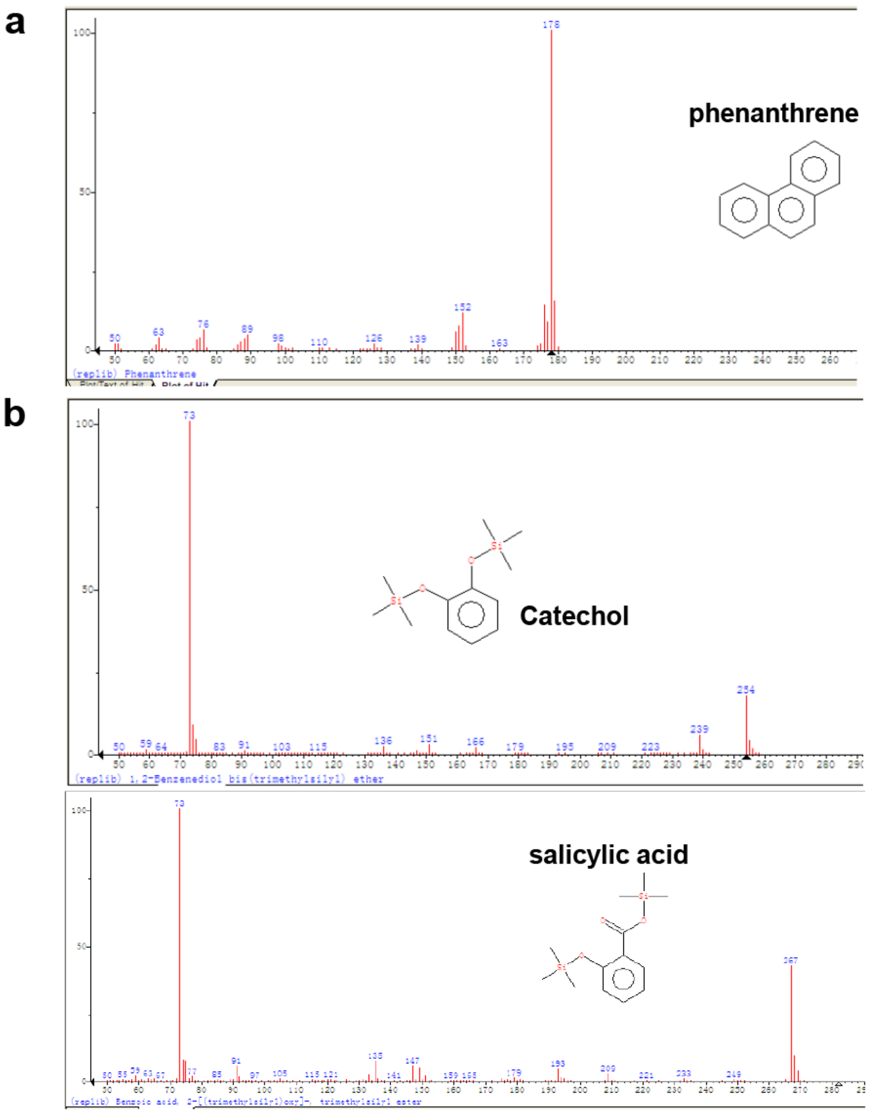

Figure S2. Mass spectra of the neutral (a) and acidic (b) metabolites of phenanthrene after degradation by PQ-2.

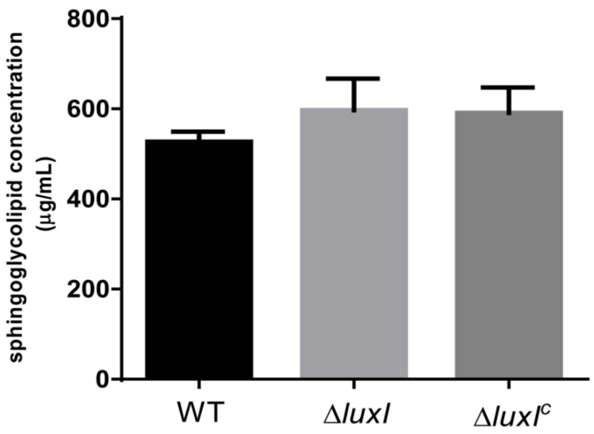

Figure S3. The content of glycosphingolipid in the wild type (PQ-2), Δ*luxI*, and its complemented strain (Δ*luxI*^{C}).
